# Low adherence to institutional clinical practice guideline in patients diagnosed with *Clostridioides difficile* infection

**DOI:** 10.1017/ash.2025.10183

**Published:** 2025-12-10

**Authors:** Zachary K. Matthews, Kayla Scheps, Joseph E. Marcus

**Affiliations:** 1 Department of Medicine, Brooke Army Medical Center, San Antonio, TX, USA; 2 Division of Infectious Diseases, Department of Medicine, Brooke Army Medical Center, San Antonio, TX, USA

## Abstract

**Objective:**

Previous antimicrobial therapy is a common risk factor for *Clostridioides difficile* infections (CDIs). Clinical practice guidelines (CPGs) assist clinicians in prescribing the correct antibiotics for various clinical syndromes to avoid unnecessary or overly broad-spectrum antibiotic use. Understanding adherence to CPGs can help determine the preventability of CDIs.

**Design:**

Quality improvement project.

**Setting:**

Tertiary military medical hospital.

**Patients or Participants:**

All patients with positive test for *Clostridioides difficile* at Brooke Army Medical between November 2022 and October 2023.

**Results:**

During the evaluation period, 128 positive cases were identified. In the three months prior to a positive test, 85 (67%) of patients had at least one day of antibiotic exposure. For those receiving antibiotics, the median number [Interquartile Range (IQR)] of total antibiotic days within 30, 60, and 90 days of a positive test was 6 [2 – 9], 7 [4.5 – 13.5], and 7 [5 – 14] days, respectively. In these cases, all antibiotic indications appeared on the CPG for 50 (59%) cases. However, prescribers did not adhere to the CPG in 23 (46%) of these cases. Overall, 58 cases (45%) were considered preventable due to clinicians not following the CPG or a condition not appearing on the CPG.

**Conclusions:**

In this single center study, retrospective analysis of individual CDI provided direction for the antimicrobial stewardship team by identifying CPG recommendations that were not followed as well as identifying conditions not on previous CPGs. Tying patient harm from CDIs with inappropriate antimicrobial use builds the local evidence base to prevent future CDIs.

## Introduction

*Clostridioides difficile* infection (CDI) is a significant cause of diarrhea in both the healthcare and community setting. CDI is most frequently associated with antibiotics that disrupt the gastrointestinal microbiota, which allows toxin-producing bacteria to replicate, leading to disease that can range from mild diarrhea to life-threatening colitis. It is estimated that there are approximately 500,000 cases and 30,000 deaths due to CDI annually in the United States.^1^ Due to the significant burden of disease, both inpatient and outpatient settings are tasked with implementing strategies to mitigate CDI and its associated risks through antimicrobial stewardship programs. As a result, the estimated burden of infection and associated hospitalizations declined from 2011 through 2017.^2^ One method utilized by antimicrobial stewardship programs to decrease CDI is to reduce inappropriate antibiotic burden for common clinical conditions through institutional clinical practice guidelines (CPG).

The concept of preventability has been successfully applied to healthcare infections to improve the implementation of known best practices and subsequently decrease the rates of infection.^3^ With limited data on the causes of CDI in military healthcare systems, it is unknown how well the concept of preventability can be applied to decreasing CDI rates. This project assessed antibiotic prescribing in patients who developed CDI and whether preceding antibiotic prescribing was in accordance with the annually updated, multi-disciplinary CPGs to determine preventability of infection.

## Methods

A retrospective analysis was performed on patients who tested positive for *C. difficile* at Brooke Army Medical Center (BAMC) between November 2022 and October 2023. Brooke Army Medical Center is the largest medical facility in the Department of Defense and provides inpatient and outpatient care to service members, families, and retirees. All stool samples that tested positive by polymerase chain reaction with Xpert C. difficile (Cepheid, Sunnyvale, CA, USA) or by Biofire Filmarray Gastrointestinal (GI) Panel (bioMerieux, Marcy-l’Étoile, France) were included in this analysis. At this center, there is no toxin confirmatory testing due to previous local studies showing minimal impact on CDI treatment decisions.^4^

**Figure 1.**
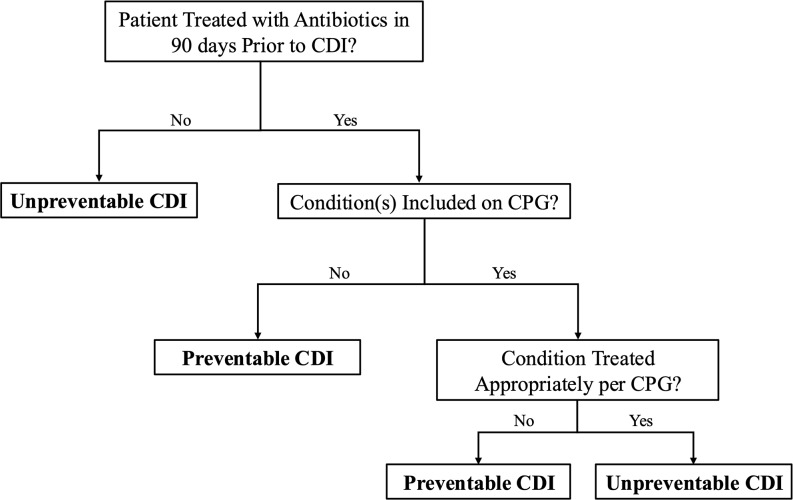
Classification of preventability. This flow diagram outlines the process used to classify infections as preventable or unpreventable. If the patient did not receive antibiotics in the 90 days prior to *Clostridioides difficile* infection (CDI), the infection was deemed unpreventable. For patients treated with antibiotics, treated conditions were first assessed for appearance on an institutional clinical practice guideline (CPG). If not included on the CPG, the CDI was determined to be preventable. If included on the CPG, adherence to the CPG was assessed. Cases without adherence to antibiotic selection recommendation were classified as preventable, whereas those with adherence were classified as unpreventable.

For each case, variables such as patient demographics, CDI treatment, documented antibiotic allergies, and antibiotic exposure within the 90 days preceding the positive test were recorded. The indication for antibiotic therapy was also reviewed in the 90 days preceding CDI diagnosis. For cases with an indication for antibiotic therapy that appeared on clinical practice guideline (CPG) recommendations, adherence was assessed through chart reviews to determine if the correct antibiotics were selected per institutional guidance. Patients were categorized as unpreventable infections if the patient had not received antibiotics in the 90 days before treatment or if all antibiotics prescribed strictly followed the CPG (Figure 1).

## Results

During the project period, there were 128 diagnoses of *C. difficile* infection at BAMC (Table 1). 65 (50.8%) cases were diagnosed in the inpatient setting, and 63 (49.2%) cases were diagnosed in the outpatient setting or Emergency Department. The median patient age was 56.5 years [Interquartile Range (IQR) 25–75.3], with a nearly equal gender distribution (47.7% male and 52.3% female). While 98 patients (76.6%) had no documented antibiotic allergies, 16 (12.5%) had documented allergies to beta-lactams and 14 (10.1%) had documented allergies to antibiotics other than beta-lactams. 102 patients (79.6%) were treated for *C. difficile* infection. The reasons for not treating included interpretation of a positive test as colonization rather than active infection (*n* = 9, 34.6%) normal gut microbiome in children under two years old (*n* = 9, 34.6%), other detected organism on multiplex PCR platform that was thought to be the causative agent to the patient’s symptoms (*n* = 8, 30.8%). The most common treatment choice was fidaxomicin, which was administered to 83 patients (64.8%), followed by oral vancomycin in 13 patients (9.4%).

**Table 1.** Patient demographics for those testing positive for *Clostridioides difficile* (*n* = 128 positive tests)

Age, median [Interquartile Range ]	56.5 [25–75.3]
Female	67 (52.3 %)
Treated for *C. difficile*	102 (79.7%)
Fidaxomicin-only	83 (64.8%)
Vancomycin-only	13 (9.4 %)
Metronidazole-only	1 (.7 %)
Vancomycin and Metronidazole	4 (3.1%)
Fidaxomicin and Bezlotoxumab	1 (.7%)
Antibiotic Allergy	
Any Antibiotic Allergy	30 (23.4 %)
Beta Lactam Allergy	16 (12.5 %)
Any Non-Beta Lactam Allergy	14 (10.9 %)
Location of Diagnosis	
Inpatient	65 (50.8%)
Outpatient	63 (49.2%)

Antibiotic exposure in the 90 days before diagnosis of CDI was systematically assessed (Table 2). Nearly two-thirds of patients (66.4%) received antibiotics within the 90 days prior to a positive *C. difficile* test. Among those exposed, inpatient-only antibiotics accounted for the majority of cases, making up 39 cases (30.5%). 28 patients (21.9%) received outpatient antibiotics only, and the remaining 18 patients (14.1%) received a combination of inpatient and outpatient antibiotics. Overall, the median [IQR] days of antibiotic therapy were 1 [0–7], 3.5 [0–9], and 5 [0–9] days within 30, 60, and 90 days of a positive test, respectively. Evaluating only those who were prescribed antibiotics demonstrated a median [IQR] of 6 [2–9], 7 [4.5–13.5], and 7 [5–14] days of antibiotics, respectively.

**Table 2.** Antibiotic exposure and timing in relation to positive *Clostridioides difficile* test

Received Antibiotics within 3 Months of Positive Test	Number (%)
Any Antibiotics	85 (66.4 %)
Inpatient and Outpatient Antibiotics	18 (14.1 %)
Outpatient Antibiotics Only	28 (21.9 %)
Inpatient Antibiotics Only	39 (30.5 %)
Antibiotic days	Median [IQR]
Within 30 days of positive test	1 [0–7]
Within 60 days of positive test	3.5 [0–8.75]
Within 90 days of positive test	5 [0–9.75]

There was limited overlap in the syndromes that were treated for inpatients and outpatients. Tables 3 and 4 report the number of times individual conditions were treated among outpatients and inpatients. Notably, many inpatients were treated for multiple conditions. For both inpatients and outpatients, urinary tract infections were a common reason for antimicrobial use (10 cases (15 %) vs 13 cases (21 %)). Other common indications for inpatient conditions were intraabdominal infections (22%), empiric sepsis (12 %), and perioperative antibiotics (12 %). In the outpatient setting, skin and soft tissue infections (13%) were the most commonly treated condition behind urinary tract infections.

**Table 3.** Conditions treated with inpatient antibiotics in the 90 days prior to a positive *Clostridioides difficile* test

Inpatient Conditions Treated	Number of Cases with this Condition	On CPG	CPG Followed
Intraabdominal infection	14	Yes	11/14 (79 %)
Urinary tract infection	10	Yes	8/10 (80 %)
Empiric Sepsis	8	Yes	8/8 (100 %)
Perioperative antibiotics	8	No	NA
Hospital/Ventilator Acquired Pneumonia	6	Yes	4/6 (67%)
Skin and soft tissue infection	6	Yes	3/6 (50%)
Community acquired pneumonia	2	Yes	1/2 (50%)
Diarrhea	2	No	NA
Trauma prophylaxis	2	No	NA
Bronchiectasis	1	No	NA
* H. pylori* infection	1	No	NA
Neutropenic Fever	1	No	NA
Orthopedic device related infection	1	No	NA

CPG, Clinical Practice Guideline.

**Table 4.** Conditions treated with outpatient antibiotics in the 90 days prior to a positive *Clostridioides difficile* test

Outpatient Conditions Treated	Number of Cases with this Condition	On CPG	CPG Followed
Urinary tract infection	13	Yes	9/13 (69%)
Skin and soft tissue infection	8	Yes	2/8 (25%)
Diverticulitis	4	Yes	3/4 (75%)
Otitis media	4	No	NA
Community acquired pneumonia	3	Yes	3/3 (100%)
Bacterial vaginosis	2	No	NA
PCP Prophylaxis	2	No	NA
Sinusitis	2	Yes	2/2 (100%)
Abdominal pain	1	No	NA
Bronchiectasis	1	No	NA
Colitis	1	No	NA
Diarrhea	1	No	NA
Giardia	1	No	NA
Hospital/ventilator acquired pneumonia	1	No	NA
Perioperative antibiotics	1	No	NA
Pharyngitis	1	Yes	0/1 (0%)

Of the 85 patients that received antibiotics prior to CDI diagnosis, whether the treated condition appeared on the CPGs and adherence to the CPG was assessed (Table 5). In 50 (58.8%) cases, all conditions for which the patient received antibiotics appeared on the CPG. 27 (54.0%) of these cases received antibiotics with complete adherence to the CPG. The most common conditions treated with inappropriate antibiotics were skin and soft tissue infections (9 cases), urinary tract infection (6 cases), intra-abdominal infections (3 cases), hospital/ventilator acquired pneumonia (2 cases), and community acquired pneumonia (1 case). Overall, 58 (45.3%) cases were considered preventable with conditions that were not on CPG or treated with an inappropriate antibiotic per the CPG, representing 68.2% of cases receiving antibiotics.

**Table 5.** Clinical practice guideline appearance and adherence among conditions treated with antibiotics in the 90 days prior to a positive *Clostridioides difficile* adherence

Appearance on BAMC Clinical Practice Guideline	Number of Cases (%)
All conditions appeared on BAMC CPG	47 (55.3 %)
Adhered to CPG	25 (53.2 %)
Did not adhere to CPG	22 (46.8 %)
One or more conditions did not appear on BAMC CPG	38 (44.7 %)

BAMC, Brooke Army Medical Center.

## Discussion

This project evaluated the antimicrobials prescribed to patients with a positive test for *C. difficile* at a large military hospital. Key findings were that most patients received antimicrobials in the 90 days before a positive test and antimicrobials chosen only matched CPG recommendations about half of the time. Based on receiving incorrect antimicrobials or antimicrobials for conditions not on the CPG, 58 (45.3%) were considered preventable CDIs. As a result of these findings, the antimicrobial stewardship team at our institution began preliminary efforts to reduce the number of preventable infections. Specifically, additional conditions, such as otitis media and sexually transmitted infections, were added to the guideline.

Previous studies have evaluated the timing of infection after antibiotics. It has been shown that both antibiotic spectrum and duration are associated with the development of CDI.^5^ Additionally, in one single-center study, the risk of CDI was higher within 3 months of antibiotics administration.^6^ In another, for every additional day of antibiotic therapy prior to index admission, the odds of subsequent CDI development increased by 12.8%.^7^ When prior outpatient and inpatient antibiotics were compared, outpatient antibiotics were more strongly associated with CDI. Interestingly, only 35% of patients with CDI in this project received outpatient antibiotics, compared to higher outpatient exposure in prior studies. As the total antibiotics ordered for inpatients and outpatients in this project is not known, the risk associated with inpatient and outpatients is unclear. However, it emphasizes the need to focus on both inpatient and outpatient settings with stewardship efforts to prevent CDI.

Many cases of CDI are presumably preventable, often arising from modifiable risk factors such as inappropriate antibiotic use or ineffective infection prevention practices. The concept of preventability has been increasingly applied to healthcare-associated infections to drive quality improvement. A recent study utilized standardized clinical scenarios to assess whether inpatient CDI cases could have been avoided with different antibiotic choices or durations.^8^ Specifically, this was accomplished by presenting stewardship experts with real and hypothetical cases and asking whether alternative management could have prevented CDI; survey results were applied to a retrospective cohort study of CDI patients with the aim of identifying the proportion of preventable and unpreventable infections.^8^ Two important preventable populations were identified: community acquired CDI in the absence of outpatient clinical practice guidelines and hospital acquired CDIs without preceding antibiotic exposure.^8^ Preventability assessments have been similarly applied to other domains, such as surgical site infections (SSI) and ventilator associated pneumonia (VAP), where bundled prevention strategies have successfully reduced rates of harm.^9,10^ At our institution, embracing preventability may help reduce overall CDI rates and to increase the proportion of cases that are deemed unpreventable.

The Military Health System (MHS) has both traditional and unique challenges when developing CDI countermeasures. On one hand, studies in the MHS show that risk factors for CDI are similar those observed in civilian healthcare settings.^11^ A study found that CDI in the MHS was associated with longer hospital stays, increased mortality, and a rising incidence from 2008 to 2015, prior to the Joint Commission’s requirement for antimicrobial stewardship teams in inpatient facilities.^11^ Unlike non-military healthcare settings, many patients are admitted due to combat trauma, which has previously been associated with the frequent antibiotic use and the development of multidrug-resistant organisms (MDROs).^12^ Given the shared risk factors between MDRO and CDI development, infection control strategies that address these factors may significantly influence CDI rates, and special attention should be considered in this domain when drafting CPGs.

There are several limitations to this single-center retrospective cohort evaluation. Importantly, our definition of preventability may overestimate inappropriate antibiotic use. Cases without conditions on the CPG were automatically classified as preventable, though some prescriber treatment options may have appropriate. Conversely, by not including duration of therapy in evaluation of CPG adherence, cases with inappropriate antibiotic use may have been missed. Next, there is a lack of differentiation between CDI and colonization, as only 80% of positive *C. difficile* tests in this cohort were treated, and this institution only does one-step testing. Further, there was no way to identify antimicrobials that were prescribed outside our health system or patients who received antimicrobials at this healthcare facility but tested positive at another hospital system. Lastly, patient factors such as comorbidities, immunosuppression, and clinical acuity are potential confounders in prescribing patterns that were not considered.

## Conclusion

This retrospective evaluation examined patients diagnosed with CDIs, focusing on antimicrobial exposure and adherence to CPGs. The results showed that a significant proportion of patients received antibiotics within the 90 days prior to CDI diagnosis. A majority of patients were considered to have preventable CDI based on inappropriate antimicrobial use or treatment for a condition not found on the CPG. Ongoing efforts to refine stewardship practices across military and civilian systems are essential for sustaining gains in CDI prevention.

## Research transparency and reproducibility

The data for this project is available upon request and can be made available publically.
